# Detection and Localization of Small Moving Objects in the Presence of Sensor and Platform Movement

**DOI:** 10.3390/s24041218

**Published:** 2024-02-14

**Authors:** Adam Cuellar, Abhijit Mahalanobis, C. Kyle Renshaw, Wasfy Mikhael

**Affiliations:** 1 Center for Research in Computer Vision, University of Central Florida, Orlando, FL 32816-8005, USA; 2 Department of Electrical and Computer Engineering, University of Arizona, Tucson, AZ 85721-0104, USA; 3 CREOL, The College of Optics and Photonics, University of Central Florida, Orlando, FL 32816-8005, USA; 4 Department of Electrical and Computer Engineering, University of Central Florida, Orlando, FL 32816-8005, USA

**Keywords:** detection, localization, infrared, moving target detection, MTI with platform metadata

## Abstract

In this paper, we address the challenge of detecting small moving targets in dynamic environments characterized by the concurrent movement of both platform and sensor. In such cases, simple image-based frame registration and optical flow analysis cannot be used to detect moving targets. To tackle this, it is necessary to use sensor and platform meta-data in addition to image analysis for temporal and spatial anomaly detection. To this end, we investigate techniques that utilize inertial data to enhance frame-to-frame registration, consistently yielding improved detection outcomes when compared against purely feature-based techniques. For cases where image registration is not possible even with metadata, we propose single-frame spatial anomaly detection and then estimate the range to the target using the platform velocity. The behavior of the estimated range over time helps us to discern targets from clutter. Finally, we show that a KNN classifier can be used to further reduce the false alarm rate without a significant reduction in detection performance. The proposed strategies offer a robust solution for the detection of moving targets in dynamically challenging settings.

## 1. Introduction

The detection of small (For the purposes of our discussion, we refer to targets with fewer than 32 pixels as “small”) moving targets using imaging sensors, when both the platform and sensor are also moving independently, is a difficult problem that has long been a subject of interest. It is well known that when the platform and sensor are both stationary, it is relatively straightforward to detect moving targets using change detection algorithms. For cases where the platform is stationary, but the sensor is moving (e.g., panning or scanning), frame-to-frame registration can be used to stabilize the video prior to applying change detection algorithms. However, when the platform is in motion, and the sensor is also moving to execute a search pattern, it become extremely challenging to discriminate between moving targets and the combined effects of the movement of the sensor and the platform. This is because even stationary objects and clutter in the foreground appear to move against the background due to parallax effects, making it difficult to distinguish them from real moving targets in such cases.

We have previously shown [1] that for stationary platforms, the Reed Xiaoli anomaly (RXA) detector (originally developed for spectral anomaly detection) can be modified to detect moving objects as temporal anomalies in sequentially registered video frames. When the sensor is moving (but the platform is stationary), the frame-to-frame registration can be improved by using sensor’s inertial data to ensure precise frame alignment. However, when the sensor and platform are moving independently, achieving accurate frame-to-frame registration becomes significantly more challenging and processing-intensive, necessitating extremely accurate measurements. In such cases, we show that a spatial anomaly detector (e.g., the Mexican Hat filter) can be used in combination with knowledge of the platform velocity and elapsed time between frames to estimate the range (or distance) of each detection. In turn, this can be used to separate stationary clutter from moving targets over multiple observations. Further reduction in false alarm rate is achieved using a KNN classifier to discriminate between potential targets and clutter objects. Cumulatively, our results shed light on the pronounced benefits of our approach in detecting small moving targets in real-world dynamic scenarios.

## 2. Background Review

While the literature is abundant with research on the problem of small moving target detection in infrared imagery, there is limited focus on methods tailored for moving platforms equipped with an independently moving imaging sensor. In the existing body of work, methods for target detection are generally divided into two primary categories: sequential detection methods and single-frame detection methods [2].

Sequential detection methods often employ a series of frames to detect targets. Classical methods like 3D-matched (directional) filtering employ track-before-detection (TBD) techniques and require a priori knowledge of target shape and velocity [3]. Techniques like 3D double directional filters and methods utilizing dual-diffusion partial differential equations have further enhanced the target energy accumulation ability [4,5]. These methods, however, rely heavily on assumptions about consistent information of targets and background between frames, making them less flexible for real-world applications [5,6,7,8].

Single-frame detection methods like TopHat filtering focus on background clutter suppression in individual frames [9,10]. Techniques based on two-dimensional least mean square adaptive filters and Probabilistic PCA have also been developed for small target detection [11,12].

One of the most recent works in this area is by Gao et al., who proposed the Infrared Patch-Image (IPI) model. IPI is unique in employing local patch construction to create a patch image where the target is sparse and the background is low rank. IPI uses the Accelerated Proximal Gradient to estimate these sparse and low-rank matrices, thus providing a finer segmentation map after employing a 1D median filter [2,13]. The method further incorporates spatial and temporal data through the Mixture of Gaussians (MoG) noise model and the Markov Random Field (MRF) for more accurate target and clutter differentiation [13].

Infrared Search and Track (IRST) methods are often designed for air- and ship-borne platforms targeting mainly airborne threats [14]. Unlike our ground-based approach for detecting small objects in cluttered terrain, these systems primarily aim at detecting unresolved point targets in aerial backgrounds. Algorithmically, while IRST methods often rely on similar principles of background suppression and clutter removal, they generally employ a different set of algorithms optimized for unresolved point targets in slowly varying background clutter.

Our work aims to fill a unique gap in the literature by focusing on small moving target detection on a ground-based moving platform with an independently moving sensor. Unlike existing IPI methods and IRST approaches, our algorithm is tailored to the specific challenges presented by ground-based scenarios and does not require any a priori assumptions or knowledge of the target shape and velocity. For our comparative analysis, we employ the IPI method as a benchmark and evaluate its performance against our proposed solutions using a MWIR dataset collected with a ground-based surveillance sensor, as will be described in Section 3.1.

### 2.1. The Reed-Xiaoli Detector

The RXA detector (which was developed by Reed and Yu for spectral anomaly detection), is modified to analyze a stack of spatially registered video frames in order to find anomalies in the temporal dimension. The RXA detector assumes that the temporal fluctuations in the background follow a Gaussian distribution and uses the Gaussian Log-likelihood Ratio Test (GLRT) to identify anomalies that have a low likelihood [15]. Specifically, the RXA score is given by

(1) $$RX\left(x_{i}\right)={\left(x_{i}-\mu \right)}^{T}\cdot K_{D\times D}^{-1}\cdot \left(x_{i}-\mu \right)\;$$

where *x* is a D×1 column vector at the *i*-th pixel location, μ is the global sample mean at that location, and *K* is the sample covariance matrix of the of the same.

Typically, the Reed–Xiaoli algorithm produces a grayscale intensity image that is usually visually inspected for detection. This is automated by applying an intensity threshold to identify and extract anomalous target pixels from the background. The grayscale image is transformed into a binary image, where targets can be detected by applying a threshold. Anomalies in the scene are expected to behave as outliers and fall into the tail of the intensity distribution.

The probability of the Reed–Xiaoli detection score, denoted as α, can be expressed as P(α). To automatically identify anomalous target pixels, a threshold, $\alpha_{0}$, is determined by setting a confidence coefficient, γ, such that $P(\alpha_{0})=\gamma$ is satisfied. Usually, γ is chosen to be the right tail of the distribution, such as 0.99 or greater, to ensure high confidence in the detection. If a pixel’s RXA score α exceeds $\alpha_{0}$, it is considered anomalous, and therefore a potential moving target.

### 2.2. Coordinate Systems and Transformations

In order to align the apparent background motion in a series of frames, registering images to a common reference frame is crucial. Image registration is facilitated by integrating GPS and INS information with the positional data from the gimbal encoders, allowing for precise tracking and comparison of the motion of Points of Interest (PoI) relative to a reference frame. Direction Cosine Matrices (DCMs), commonly used in integrated navigation systems, are utilized to determine the position of points in the reference frame by translating vector components from one coordinate frame to another [16].

To align the moving sensor frame, *M*, to the fixed sensor frame, *F*, we use the azimuth and elevation angles of the gimbal at frame *M* to create a transformation matrix, $R_{S_{M}}^{G}$, that relates components from the sensor frame *M* to the gimbal frame. This relationship is given by the equation

(2) $$\vec{U^{G}}=R_{S_{M}}^{G}\cdot \vec{U^{S_{M}}}$$

The inverse of the transformation matrix from the fixed sensor frame *F* to the gimbal frame, $R_{S_{F}}^{G}$, is used to find the rotation matrix from the gimbal frame to the fixed sensor frame *F*, $R_{G}^{S_{F}}$. This is represented by:

(3) $$R_{G}^{S_{F}}={\left(R_{S_{F}}^{G}\right)}^{-1}\;$$

These matrices allow us to relate each point in frame *M* to a point in frame *F* by transforming the corresponding unit vector.

We calculate a homography matrix describing the transformation between the two images by selecting four points in frame *M*, transforming their corresponding unit vectors to frame *F*, and calculating the perspective transform using the pixel locations of these points in *M* and frame *F*. The points are chosen to be $\pm \frac{1}{4}$ the image width and $\pm \frac{1}{4}$ the image height from the image center. The corresponding unit vectors are found using the azimuth α and elevation β from the sensor’s field of view and the center vector, ${\vec{U}}_{C}=\left[1,0,0\right]$. Given these angles, the vectors are rotated using the consolidated matrix

(4) $$R=\left[\begin{array}{ccc} \cos(\alpha )\cos(\beta ) & -\sin(\alpha ) & \cos(\alpha )\sin(\beta )\\ \sin(\alpha )\cos(\beta ) & \cos(\alpha ) & \sin(\alpha )\sin(\beta )\\ -\sin(\beta ) & 0 & \cos(\beta ) \end{array}\right]$$

to derive the rotated vector ${\vec{U}}^{R}=R\cdot {\vec{U}}^{C}$. It is important to note that this matrix *R* is valid only under the condition that there is no roll in the gimbal, aligning with the specific configuration of the gimbal in our system. To obtain the pixel representation of these points, each vector is converted to a camera-relative line-of-sight vector, which is then transformed into pixel space using the intrinsic parameters of the camera. During this transformation, radial and tangential distortions are applied as follows:

(5) $$x_{d}=x\left(1+k_{1}r^{2}+k_{2}r^{4}+k_{3}r^{6}\right)+\left[2p_{1}xy+p_{2}\left(r^{2}+2x^{2}\right)\right]$$

(6) $$y_{d}=y\left(1+k_{1}r^{2}+k_{2}r^{4}+k_{3}r^{6}\right)+\left[p_{1}\left(r^{2}+2y^{2}\right)+2p_{2}xy\right]$$

where $r^{2}=x^{2}+y^{2}$, $k_{1},k_{2},k_{3}$ are the radial distortion coefficients and $p_{1},p_{2}$ are the tangential distortion coefficients. Finally, the original points from frame *M* and the distorted points in frame *F* are used to calculate the perspective transformation matrix.

The process described aligns two consecutive image frames obtained with a moving sensor. To include cases where the platform is also moving, a different reference frame must be considered. The gimbal frame is used as the reference when only the sensor is moving, but the Earth-Center, Earth-Fixed (ECEF) coordinate system must be used as the reference frame to take platform motion into account. To facilitate this, we use several rotation matrices:

- $R_{G}^{B_{M}}$: This matrix represents the rotation from the gimbal frame to the moving base frame, describing how the gimbal is oriented relative to the moving platform at a specific instance.
- $R_{B_{M}}^{ECEF}$: This matrix translates the moving base frame to the ECEF coordinate system, accounting for the platform’s position and orientation on Earth.
- $R_{ECEF}^{B_{F}}$: This matrix is the rotation from the ECEF coordinate system to the fixed base frame, relative to a non-moving base frame.
- $R_{B_{F}}^{G}$: This matrix describes the rotation from the fixed base frame to the gimbal frame, indicating how a stationary platform would be oriented relative to the gimbal.
- $R_{G}^{S_{F}}$: As previously mentioned, this matrix transforms from the gimbal frame to the fixed sensor frame, accounting for the sensor’s orientation relative to a fixed gimbal.
- $R_{S_{M}}^{G}$: This matrix describes the rotation from the sensor frame in the moving frame to the gimbal frame, which is crucial for understanding the orientation of the sensor relative to the gimbal when both are in motion.

By employing these matrices, we can accurately describe the rotations between various frames. The transformation from the moving sensor frame component $\vec{U^{S_{M}}}$ to the fixed sensor frame component $\vec{U^{S_{F}}}$, considering both sensor motion and platform motion, is then given by the comprehensive equation

(7) $$\vec{U^{S_{F}}}=R_{G}^{S_{F}}\cdot R_{B_{F}}^{G}\cdot R_{ECEF}^{B_{F}}\cdot R_{B_{M}}^{ECEF}\cdot R_{G}^{B_{M}}\cdot R_{S_{M}}^{G}\cdot \vec{U^{S_{M}}}$$

**Figure 1:**
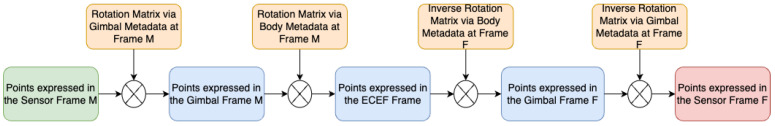
Diagram of the process of rotating points from a fixed frame M to a moving frame F using Direction Cosine Matrices.

This approach ensures a thorough consideration of all the rotational influences affecting the sensor and the platform, thus enabling a more precise image registration process. An overview of the approach is shown in Figure 1.

## 3. Materials and Methods

### 3.1. Data Collection Methodology

**Figure 2:**
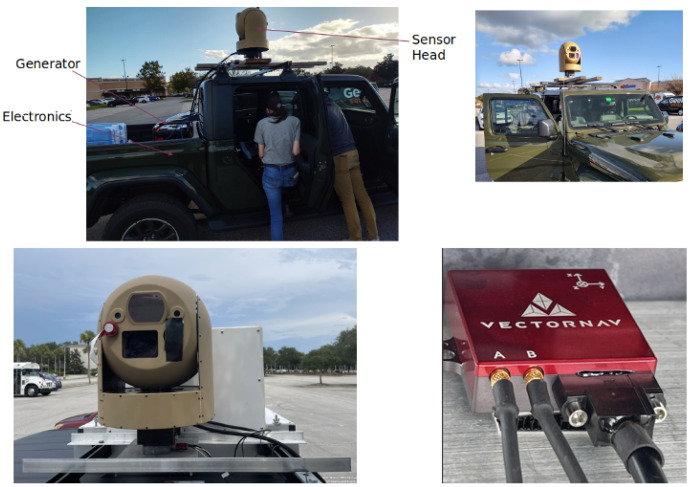
Data collection setup. (**Top Left**) Setup of the surveillance imaging sensor on the Jeep. (**Bottom Left**) Final placement of the VectorNav and the imaging sensor. (**Bottom Right**) VectorNav and its orientation.

For the purposes of our study, we collected 30 Hz video using a MWIR imaging sensor mounted on top of a 2022 Jeep Gladiator, along with an inertial measuring unit, the VectorNav VN-300, directly beneath the sensor. The video frames are of size 640 × 512. The VN-300 is a sophisticated Dual Antenna GNSS-Aided Inertial Navigation System, which provides optimal estimates of position, velocity, and orientation. It uses two separate GNSS receivers and antennas, enabling accurate heading measurements without relying on the vehicle dynamics or magnetic sensors [17]. Our data collection setup was specifically designed with the VectorNav placed as close to the sensor as possible in order to minimize the displacement between the sensor frame and body frame. The setup is shown in Figure 2.

**Figure 3:**
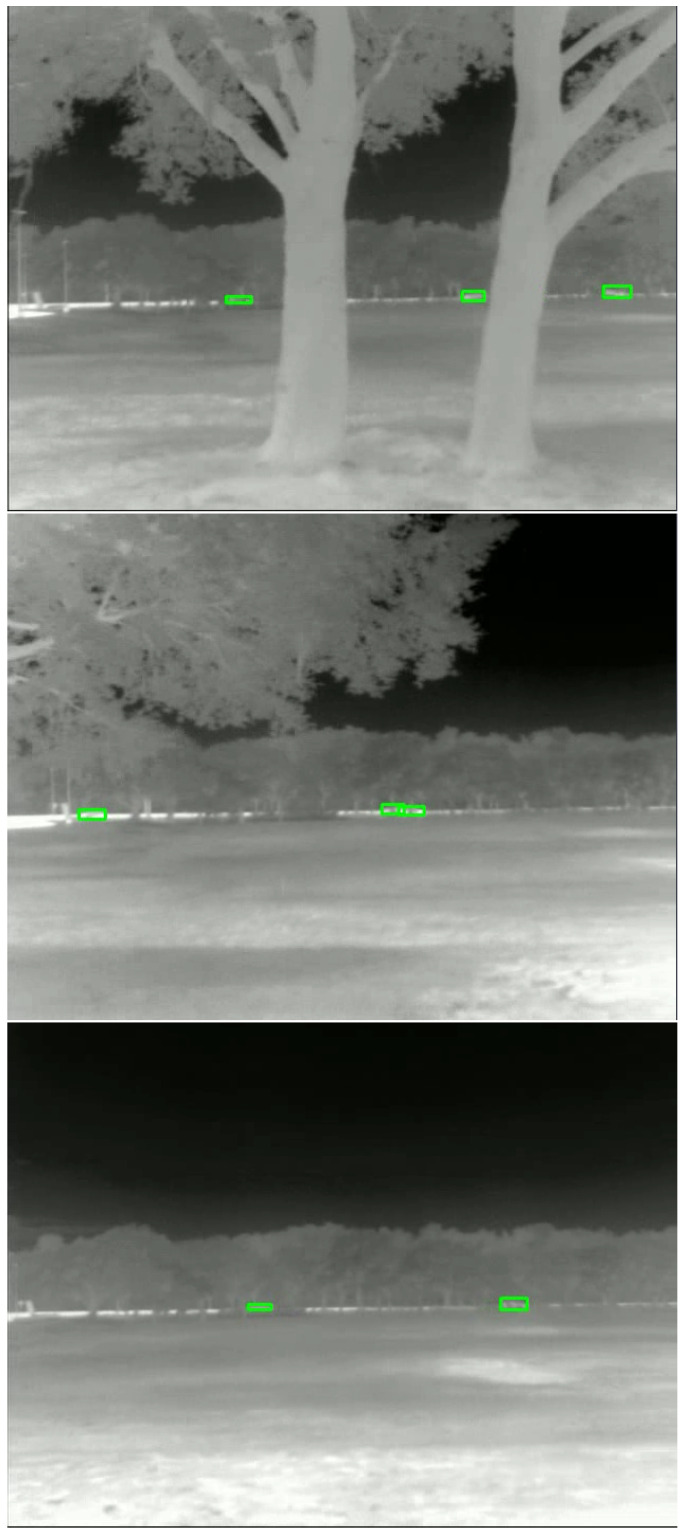
Illustration of successive frames obtained from the data collection phase where the platform and sensor were both in motion. (**Top**) First frame. (**Middle**) Second frame. (**Bottom**) Third frame. Green bounding boxes indicate the presence of targets.

We first collected data for the scenario in which both the sensor and platform were stationary. These data were used to evaluate the algorithms under ideal conditions when no sensor and platform motion are present. We then collected data for the scenario in which the platform was stationary while the sensor was panning. The sensor was moved at a constant speed from right to left and then back again. Additionally, for the cases in which the platform is in motion, the vehicle was kept at a constant velocity and was driven on a flat road. This setup was used both when the sensor was kept stationary and for the scenario where both the sensor and platform were in motion, simulating a real-world use case. Figure 3 displays an example of consecutive frames from the data collection scenario where both the platform and sensor were in motion. The green boxes in the figure indicate the location of the moving targets in the scene.

The metadata collected from the VectorNav VN-300 are crucial for incorporating inertial data into the image alignment process and determining range information. These metadata include time, azimuth, elevation, and velocity of the sensor’s position in the Local Level North-East-Down frame, as well as the yaw, pitch, and roll of the platform. By utilizing Kalman filters, the VectorNav VN-300 provides accurate measurements over time, allowing for precise image alignment and range estimation during each of the diverse real-world scenarios described previously.

A total of 24 videos were collected, each containing approximately 2–3 targets throughout the duration of the video. These videos were collected under a variety of lighting conditions, encompassing both day and night scenarios, specifically in favorable weather conditions void of rain, fog, or other adverse elements. With 15 videos recorded during the day and 9 at night, the platform and sensor configurations for the videos varied: 10 were recorded with a stationary platform and moving sensor, 6 with both the platform and sensor stationary, and the remaining 8 with both the platform and sensor moving.

Before utilizing the collected data, it is crucial to accurately calibrate the sensor system. This calibration process involves determining the intrinsic parameters of the camera, such as the focal length and image center, as well as the extrinsic parameters, such as the position and orientation of the camera relative to the platform. By calibrating the sensor, we can ensure accurate reprojection of the inertial data into pixel space, allowing us to effectively align the images and use the inertial data to improve the performance of the proposed algorithms. This is a crucial step in the data analysis process and must be performed with care to ensure the validity of the results.

**Figure 4:**
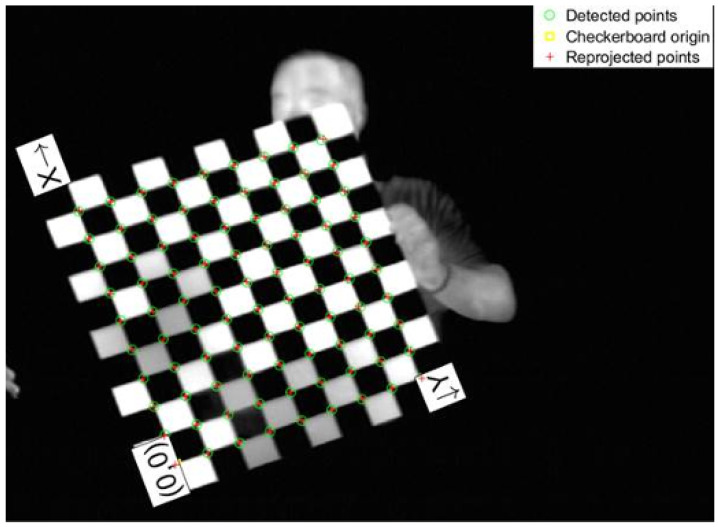
Image of a checkerboard captured with the surveillance imaging sensor. Details include calibration, detected points, origin, and reprojected points.

The calibration measurements were performed using MATLAB’s (R2022a) camera calibration tool after acquiring approximately 600 frames of a checkerboard surface with the infrared imaging sensor [18]. To ensure optimal results, a checkerboard pattern was created using black anodized metal and aluminum tape, which was attached to a rigid wooden surface. The dimensions of the checkerboard were approximately 47.6 mm in both height and width. The checkerboard setup is shown in Figure 4.

### 3.2. Proposed Approach

**Figure 5:**
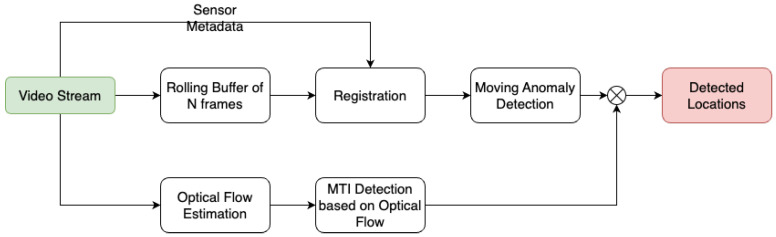
Illustration of the Moving Target Indicator with RXA.

The end-to-end approach for detecting and tracking moving targets in imagery captured by a sensor in motion is illustrated in Figure 5. We have introduced two distinct yet complementary strategies. The first is an efficient Moving Target Indicator (MTI) algorithm that relies on the RXA detector, enhanced with sensor metadata and optical flow to mitigate false alarms, which was used only for the stationary platform cases. The second, a method that differentiates between actual moving targets and stationary clutter by leveraging the platform’s motion and the camera parameters, was used when the platform was also moving.

#### 3.2.1. Moving Target Detection with RXA (Stationary Platform Case)

**Figure 6:**
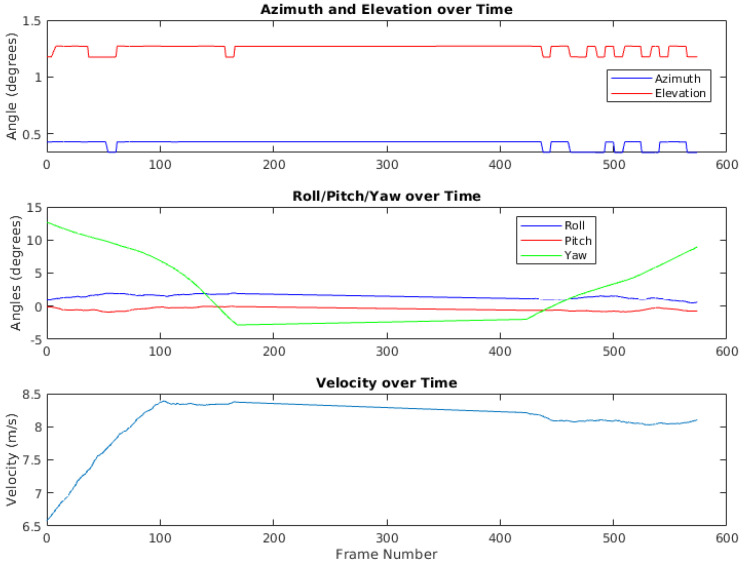
Azimuth, elevation, roll, pitch, yaw, and velocity trends for a selected video sequence.

**Figure 7:**
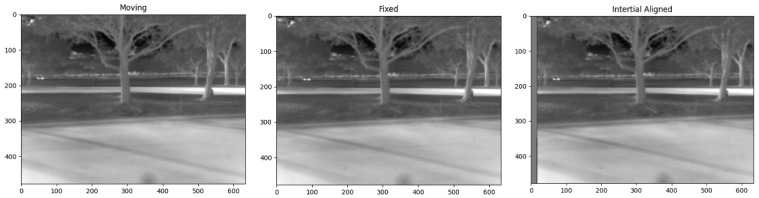
Inertial data-based registration results. (**Left**) Frame before registration. (**Middle**) Fixed reference frame. (**Right**) Frame post-registration.

The core of the first approach is the RXA detector, which, while effective for moving target indication, sometimes falters due to frame registration errors, especially in the presence of motion blur. To counteract this, we use inertial measurements and GPS data for frame alignment. Figure 6 shows an example of the inertial measurements and GPS data. As reported in our previous work [1], we stack *N* consecutive frames before executing the detection algorithm, aligning each with the central frame in the stack using the methods detailed in Section 2.2. Using the inertial metadata, we fetch the necessary rotation matrices to transition points in moving frames to the fixed center frame via direction cosine matrices. After rotating these points, we conduct a perspective transformation to align images to a common reference. For enhanced registration, we also use feature-based registration methods. An example of the registration results is shown in Figure 7.

**Figure 8:**
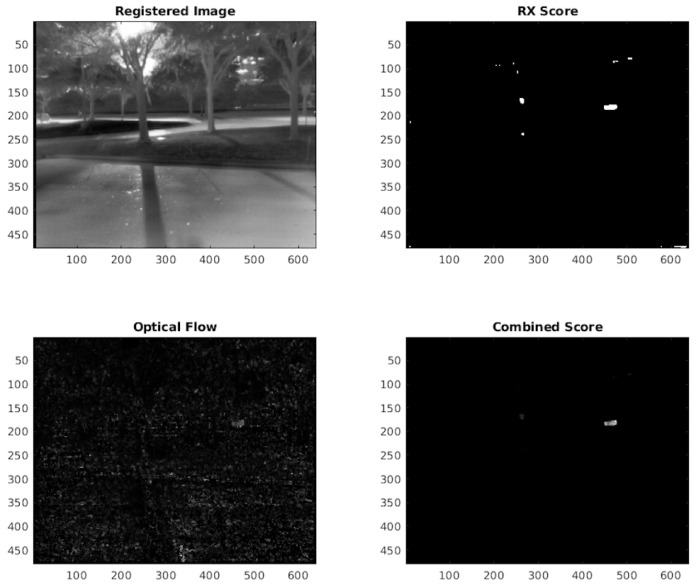
Visual results of combining the optical flow response and the Reed–Xiaoli response after image registration.

Upon stabilizing the sequence of frames, the RXA detector, as previously elaborated, can be employed. A thresholding technique as mentioned in Section 2.1 allows the RXA detector to manage a variable number of detections, but may lead to more false alarms. To handle this, we compute each pixel’s optical flow and use it to further fine-tune the RXA output. Specifically, each pixel’s output from the RXA is multiplied by its optical flow vector’s magnitude, helping to reduce false alarms, especially when optical flow magnitudes are low. This method is visualized in Figure 8.

#### 3.2.2. Advanced Moving Target Discrimination (Moving Platform Case)

**Figure 9:**
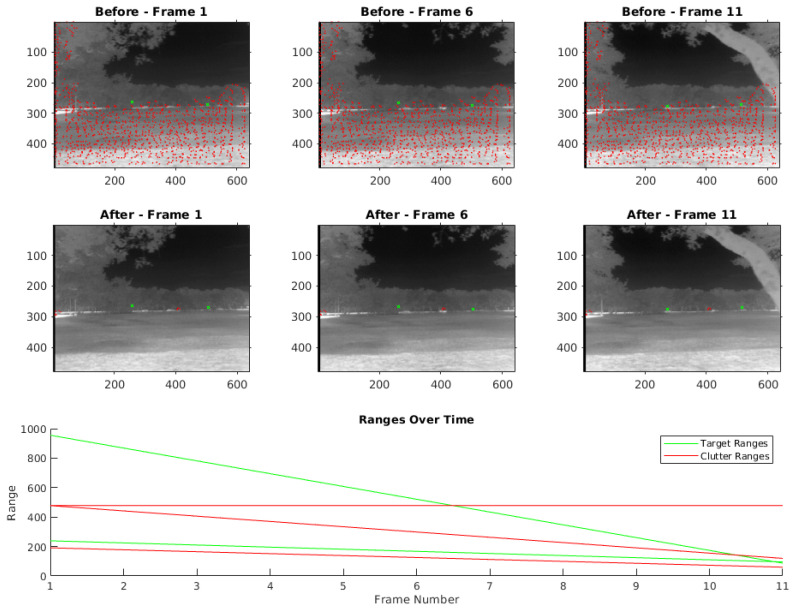
Frames illustrating target and clutter detection using the Mexican Hat filter and post-filtering methods.

Our strategy for efficient moving target detection and clutter discrimination harnesses both spatial and temporal techniques. To leverage temporal information, we work with sets of three frames, with several frames skipped in between. Initially, we set the number of skipped frames to five, but it can be adjusted according to specific requirements. We first utilize a Mexican Hat filter for the initial detection in each of the selected frames, optimizing the kernel to respond to small localized variations in intensity, which often signify potential moving targets. A visual representation of this can be observed in the top row of Figure 9, where three frames from the video sequence are shown. Targets are marked in green and clutter in red.

Post application of the Mexican Hat filter, we exploit camera parameters and the platform’s velocity to examine points across the multiple frames, subsequently determining the point’s range over time. The bottom row of Figure 9 visualizes the range of the detections throughout the frame sequence, highlighting that while the range of the targets varies, the clutter maintains more consistent range values. By contrasting the discrepancy between the calculated range over time, we can discern whether a point is a moving target or clutter. Points with inconsistent range shifts are flagged as targets, while those with linear range alterations paralleling their apparent motion are deemed stationary clutter.

For range estimation, we tap into the linear motion between subsequent frames, likening it to a series of stereo images. We thus determine the range in a manner akin to stereo image processing using the equation

(8) $$r=\frac{v\cdot t}{iFOV\cdot \sqrt{{\left(x_{2}-x_{1}\right)}^{2}+{\left(y_{2}-y_{1}\right)}^{2}}}$$

Here, *r* denotes the estimated range, *v* represents the platform’s velocity, *t* is the time difference between frames, iFOV indicates the sensor’s instantaneous field of view, while $\left(x_{1},y_{1}\right)$ and $\left(x_{2},y_{2}\right)$ specify the pixel coordinates in two successive frames.

**Figure 10:**
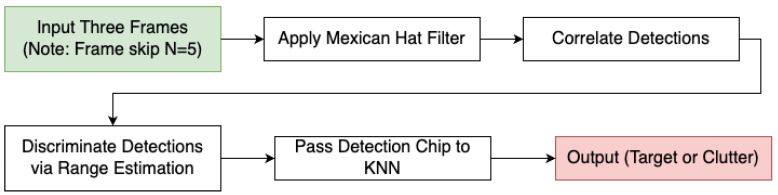
Advanced moving target discrimination using Mexican Hat filter and range estimation.

**Figure 11:**
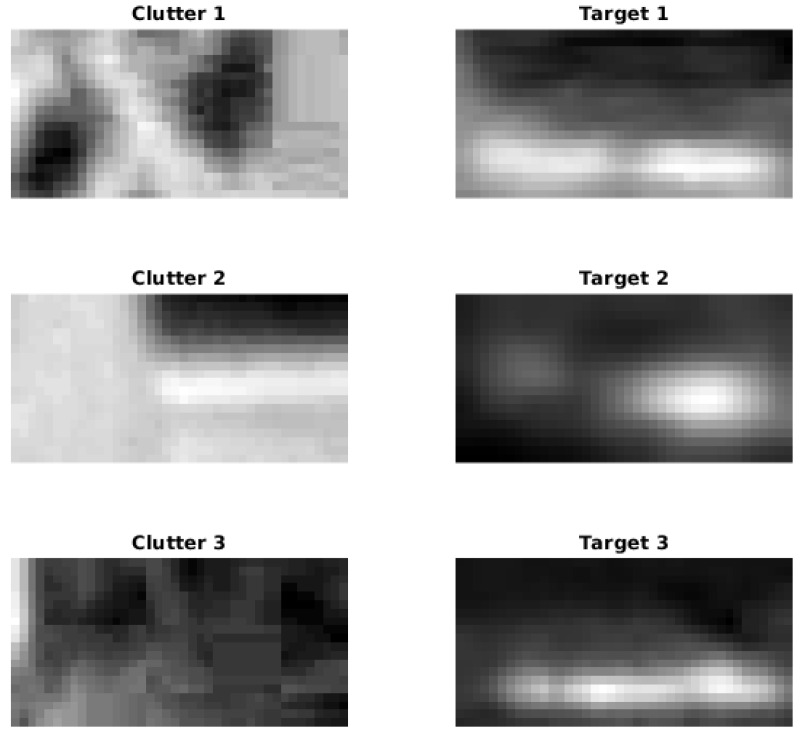
Example image chips used for training the KNN classifier. (**Left**) Clutter examples. (**Right**) Target examples.

To further refine the discrimination process and reduce the chances of misclassification, we introduce a KNN classifier that is trained on different frames from those we evaluate. This ensures that our classifier is robust, can generalize well to new data, and substantially reduces false alarms by leveraging patterns and relationships learned during the training phase. Notably, the KNN model achieved a 98.49% accuracy on the training data, indicating its high effectiveness in identifying target characteristics accurately. The resulting filtered detections can be seen in the middle row of Figure 9. An illustrative outline of the process described above is provided in Figure 10. Example image chips used to train the KNN classifier are shown in Figure 11.

## 4. Results

In our experiments, various methods of moving target indication and discrimination were analyzed and compared. The Infrared Patch-Image (IPI) model exhibited disappointing performance on the dataset collected with the surveillance imaging sensor, even when both the sensor and platform were stationary. Due to its evident inefficacy in these scenarios, we decided against employing the IPI algorithm in situations with different sensor and platform movements.

**Figure 12:**
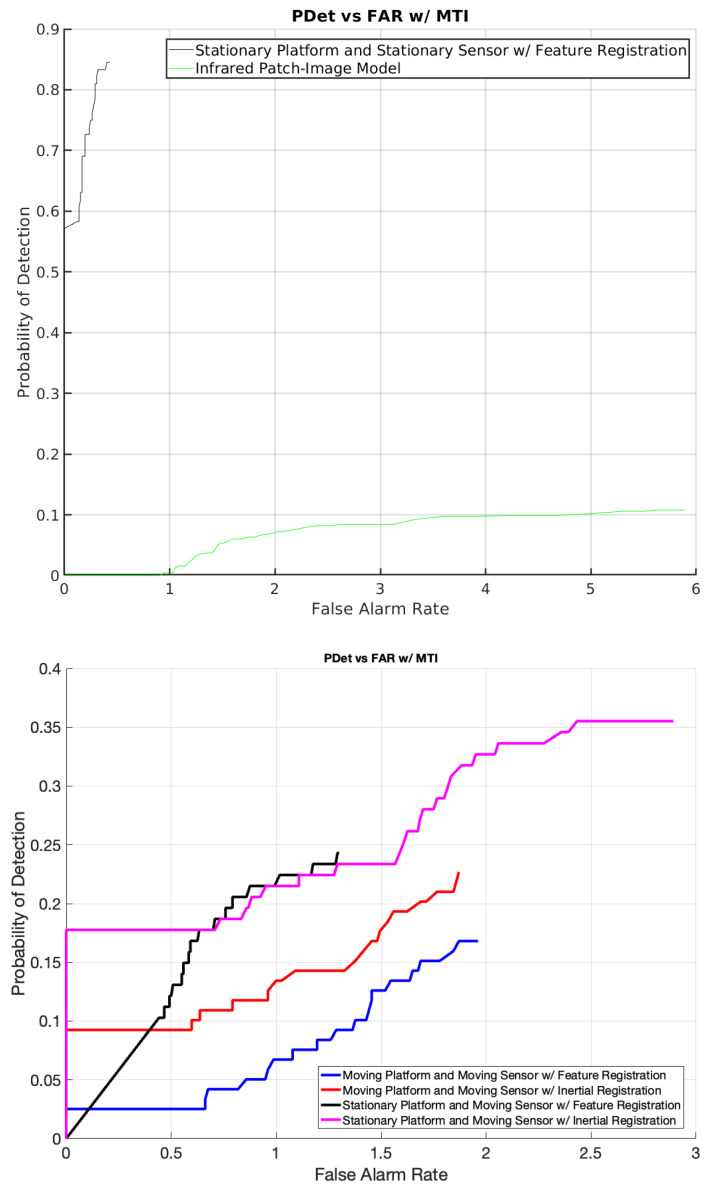
(**Top**) Performance comparison of the Infrared Patch-Image (IPI) model against the proposed MTI algorithm with feature-based registration when the sensor and platform are stationary. (**Bottom**) Results of the MTI approach under varying motion scenarios.

Our proposed MTI approach with inertial-based registration was compared against traditional feature-based registration. The baseline results for scenarios with a stationary sensor and platform are depicted in part A of Figure 12. The ROC curves illustrate a robust detection probability with a well-controlled false alarm rate. However, feature-based registration encountered difficulties as the movement in the sensor and/or platform escalated, a limitation somewhat alleviated by the introduction of inertial registration.

**Figure 13:**
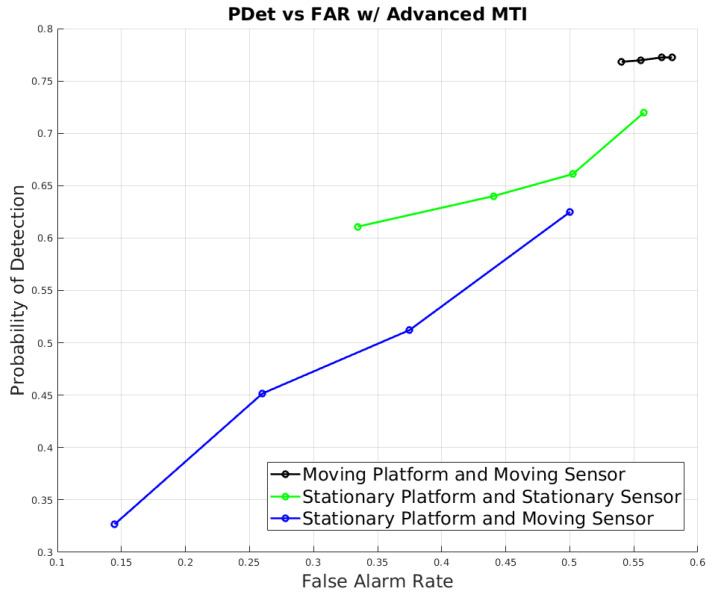
Performance results of the advanced moving target discrimination method across different scenarios.

For the advanced target discrimination method (for moving platforms), the Mexican Hat filter was employed for initial detection, coupled with range estimation and KNN discrimination for enhanced accuracy across various scenarios, as evident from Figure 13. Range estimation was pivotal only in scenarios in which the platform was in motion, owing to the necessity of a non-zero platform velocity for accurate range calculations. In stationary platform scenarios, feature-based registration was leveraged to discern difference images between frames, which were then processed by the Mexican Hat detector due to its capability to identify targets with reduced false alarms. However, the performance of feature-based registration diminished with sensor movement, causing a decrease in the probability of detection in scenarios with a stationary platform and moving sensor, as depicted in Figure 13.

When both the platform and sensor were in motion, range estimation played a crucial role in discriminating between targets and clutter, significantly mitigating the false alarms produced by directly applying the Mexican Hat detector to the image frames. This adaptive approach and the subsequent KNN discrimination collectively enhanced detection probabilities and notably curtailed false alarms in different operational contexts.

For the construction of the ROC curve in Figure 13, varying thresholds were applied for range variation to discriminate between targets and clutter in scenarios involving moving platforms and sensors, evidencing the significant strides made by this method in bolstering detection capabilities. Conversely, for the scenarios involving stationary platforms, where range estimation was not applicable, we modified the number of detections permitted by the Mexican Hat detector to create varying degrees of sensitivity, allowing for a balanced and nuanced comparative analysis across diverse operational settings.

## 5. Conclusions

Our experiments highlighted the inherent complexities and challenges in moving target indication and discrimination under varying conditions. The Infrared Patch-Image (IPI) model proved unsatisfactory, specifically in stationary scenarios, necessitating exploration of more robust alternatives.

Our proposed MTI method with inertial-based registration demonstrated superior reliability and effectiveness compared to the traditional feature-based registration, especially in scenarios involving increased movement of sensors and/or platforms.

The integration of the Mexican Hat filter, range estimation, and KNN discrimination in our advanced method displayed enhanced performance across diverse scenarios, resulting in improved detection probabilities and a reduction in false alarms. This underscores the value of a multifaceted approach to moving target detection in dynamic environments.

Our approach to the problem provided unique insights into the intricate dynamics of moving target detection. The adaptive utilization of different methods, depending on the movement of the platform and sensor, addressed the specific challenges posed by different operational contexts.

While this research makes advancements in moving target detection and discrimination, it also opens up avenues for future exploration, particularly in the realm of adverse weather conditions. The effect of factors like data dropouts, equipment malfunction, and noise in measurements during such conditions is a topic for future research. This consideration is crucial as it represents a realistic aspect of operational environments that could impact the effectiveness of current methodologies.

This research provides insights into the potential of combining different techniques to enhance moving target indication in dynamic scenarios and lays a foundational framework for future research. The findings encourage the refinement and integration of spatial and temporal techniques to better address evolving challenges in the field of moving target detection and discrimination.

## Data Availability

The data presented in this study are available on request from the corresponding author.

## Abbreviations

The following abbreviations are used in this manuscript:

GPS	Global Positioning System
INS	Inertial Navigation System
DCM	Direction Cosine Matrix
ECEF	Earth-Centered, Earth-Fixed
GNSS	Global Navigation Satellite System
MWIR	Medium-Wave Infrared
IRST	Infrared Search and Track
GLRT	Gaussian Log-likelihood Ratio Test
IPI	Infrared Patch-Image
MoG	Mixture of Gaussians
MRF	Markov Random Field
MTI	Moving Target Indicator
RXA	Reed Xiaoli Anomaly
KNN	K-Nearest Neighbors
ROC	Receiver Operating Characteristic
TBD	Track Before Detection
PCA	Principal Component Analysis
VN-300	VectorNav VN-300 (specific model of an inertial measuring unit)

## References

[1] Cuellar A., Mahalanobis A. Detection of Small Moving Ground Vehicles in Cluttered Terrain Using Infrared Video Imagery. Proceedings of the 2021 IEEE International Conference on Image Processing (ICIP).

[2] Gao C., Meng D., Yang Y., Wang Y., Zhou X., Hauptmann A.G. (2013). Infrared Patch-Image Model for Small Target Detection in a Single Image. IEEE Trans. Image Process..

[3] Reed I.S., Gagliardi R.M., Stotts L.B. (1988). Optical moving target detection with 3-D matched filtering. IEEE Trans. Aerosp. Electron. Syst..

[4] Li M., Zhang T., Yang W., Sun X. (2005). Moving weak point target detection and estimation with three-dimensional double directional filter in IR cluttered background. Opt. Eng..

[5] Liu X., Zuo Z., Tan T., Ruan Q., Chen X., Ma H., Wang L. (2013). A Dim Small Infrared Moving Target Detection Algorithm Based on Improved Three-Dimensional Directional Filtering. Proceedings of the Advances in Image and Graphics Technologies.

[6] Porat B., Friedlander B. (1990). A frequency domain algorithm for multiframe detection and estimation of dim targets. IEEE Trans. Pattern Anal. Mach. Intell..

[7] Zhang B., Zhang T., Cao Z., Zhang K. (2007). Fast new small-target detection algorithm based on a modified partial differential equation in infrared clutter. Opt. Eng..

[8] Wei P., Zeidler J., Ku W. (1995). Analysis of multiframe target detection using pixel statistics. IEEE Trans. Aerosp. Electron. Syst..

[9] Tom V.T., Peli T., Leung M., Bondaryk J.E. Morphology-based algorithm for point target detection in infrared backgrounds. Proceedings of the Signal and Data Processing of Small Targets 1993.

[10] Toet A., Wu T. Small maritime target detection through false color fusion. Proceedings of the Optics and Photonics in Global Homeland Security IV.

[11] Soni T., Zeidler J.R., Ku W.H. (1993). Performance evaluation of 2-D adaptive prediction filters for detection of small objects in image data. IEEE Trans. Image Process..

[12] Cao Y., Liu R., Yang J. (2008). Small target detection using two-dimensional least mean square (TDLMS) filter based on neighborhood analysis. Int. J. Infrared Millim. Waves.

[13] Gao C., Wang L., Xiao Y., Zhao Q., Meng D. (2018). Infrared small-dim target detection based on Markov random field guided noise modeling. Pattern Recognit..

[14] Du P., Hamdulla A. (2020). Infrared Moving Small-Target Detection Using Spatial–Temporal Local Difference Measure. IEEE Geosci. Remote Sens. Lett..

[15] Reed I., Yu X. (1990). Adaptive multiple-band CFAR detection of an optical pattern with unknown spectral distribution. IEEE Trans. Acoust. Speech Signal Process..

[16] Rogers R.M. (2003). Coordinate Systems and Transformations. Applied Mathematics in Integrated Navigation Systems.

[17] VectorNav VN-300. https://www.vectornav.com/products/detail/vn-300.

[18] MATLAB Camera Calibration. https://www.mathworks.com/help/vision/camera-calibration.html.

